# Brugada Syndrome: an exemplar for the genomic basis of sudden death

**DOI:** 10.1038/s41431-025-01972-0

**Published:** 2025-11-14

**Authors:** Rebecca L. M. Griffiths, Roddy Walsh, Marta Futema, Mark Specterman, Elijah R. Behr

**Affiliations:** Cardiovascular and Genomics Research Institute, School of Health and Medical Sciences, Tooting Campus, City St George’s, University of London, Cranmer Terrace, London, SW17 0RE UK

**Keywords:** Disease genetics, Medical genetics, Arrhythmias, Genetics research, Genome-wide association studies

## Abstract

The inherited arrhythmia syndrome, Brugada Syndrome (BrS), is a leading cause of autopsy negative sudden death: the sudden arrhythmic death syndrome. Historically, BrS was believed to exhibit a Mendelian (autosomal dominant) mode of inheritance, caused by rare variants in *SCN5A*, the gene coding for the alpha subunit of the main cardiac sodium voltage channel. Challenges to this paradigm have arisen. For example, the majority of BrS cases do not exhibit rare variants in *SCN5A*. Moreover, genotype-phenotype mismatch in families has been observed. These findings suggest a more complex genetic architecture underpinning BrS. Subsequent large genomic studies of international patient cohorts have shown an unexpectedly high contribution of common genetic variation to its phenotypic development and severity. This has led to an alternative disease hypothesis whereby BrS develops as result of accumulated genetic and environmental risk surpassing a ‘disease threshold’ – the higher the accumulated risk, the more severe the clinical phenotype. Whilst expansion of standard clinical genetic testing to include an assessment of common variation might assist with diagnosis and phenotypic severity prediction in BrS, its incorporation into clinical practice presents inherent challenges which require careful consideration.

## Introduction

Sudden cardiac death (SCD) is defined as the unexpected, natural, and sudden death of a victim of a presumed cardiac aetiology, occurring within 1 hour from the onset of the victim’s symptoms (if the death is witnessed) or occurring within 24 hours of the last time the victim was seen alive (if unwitnessed) [1]. SCDs are responsible for around half of all deaths from a cardiovascular cause [2]. Fifty percent of these deaths are the first manifestation of cardiac pathology in victims [3]; predicting those at risk of SCD and employing protective strategies to avoid this devastating event (e.g., through placement of an implantable cardioverter defibrillator, ICD) is therefore of paramount importance.

To determine the underlying cause of death, all victims of SCD should undergo comprehensive autopsy, part of which includes a thorough assessment of the victim’s heart [4]. Examination of the heart by a specialist cardiac pathologist has been shown to be superior to that of a non-specialist pathologist, with the latter group tending to over-diagnose cardiac muscle diseases (cardiomyopathies) [5]. Inherited cardiac conditions (ICCs) account for almost half of SCD cases in those aged fifty years or below, examples of which can be detected at autopsy include the hypertrophic, dilated, and arrhythmogenic right ventricular cardiomyopathies [1].

Should a comprehensive autopsy and a toxicological analysis fail to reveal a cause of death, the SCD is attributed to sudden arrhythmic death syndrome (SADS). Deaths attributable to SADS have been shown to underlie almost a third of cases of cardiac deaths in the under 35-year-old age group, with young males being at a two-fold increased risk compared to their female counterparts [6]. Identification of the underlying cause is key for prevention of similar occurrences within families. Post-mortem genetic testing of the victim for a wide panel of genes (the so-called ‘molecular autopsy’) is recommended for further investigation. Genes analysed include those known to cause cardiomyopathies (as in some cases the arrhythmic event precipitating death can precede the development of structural changes that can be detected at autopsy – a ‘concealed’ cardiomyopathy [7]) and primary electrical diseases, also known as inherited arrhythmia syndromes (IASs). The molecular autopsy alone provides an underlying genetic explanation in 13% of SADS cases from all age groups [8]. Concurrently, family members of victims should be screened clinically for any suspicious symptoms, signs or investigatory findings that would support an underlying diagnosis of an ICC. Diagnostic yield improves to 39% when information from familial clinical screening is incorporated into assessments and is therefore recommended by the European Society of Cardiology and other bodies [8].

A variety of diseases are categorised as IASs, including Long QT Syndrome (LQTS), Catecholaminergic Polymorphic Ventricular Tachycardia (CPVT), Short QT Syndrome (SQTS), and Brugada Syndrome (BrS). They have classically been thought of as being inherited in an autosomal dominant manner with variable penetrance, resulting in a wide spectrum of phenotypic variation in families [9].

Clinical genetic testing aims to identify (likely) pathogenic variants in genes known to be responsible for IASs. However, its yield varies between diseases. For example, in LQTS, the diagnostic yield of genetic testing is as high as 80%, but in BrS, it is only around 20% [10–12]. As BrS accounts for a quarter of SADS cases, understanding its complex genetic architecture is of great importance for identifying those at risk of developing the disease and those most at risk of sudden death [13] – this information can then be used to inform patients’ clinical management.

## Brugada Syndrome

In the 1980s, an unusually high frequency of nocturnal SCD was documented in young, predominantly male, and otherwise healthy Southeast Asian refugees living in the United States [14, 15]. These deaths were originally ascribed to sudden unexplained death syndrome (SUDS), the phenomenon having previously been observed by locals in Northeast Thailand and Japan and described as ‘Lai Tai’ and ‘Pokkuri’ respectively [14, 15]. Meanwhile, a characteristic pattern on an electrocardiogram (ECG) had been found to associate with sudden death, this association later came to be coined ‘Brugada Syndrome’ after Pedro and Josep Brugada [16–19]. Further work established the equivalence of SUDS to BrS, with deaths caused by unheralded ventricular arrhythmias, specifically polymorphic ventricular tachycardia (pVT) and ventricular fibrillation (VF), occurring during rest and recovery [15–18, 20]. The worldwide prevalence of BrS has been shown to be approximately 0.05%, with a higher prevalence recorded in Asian populations, particularly in those from Southeastern and Eastern portions [21].

**Fig. 1:**
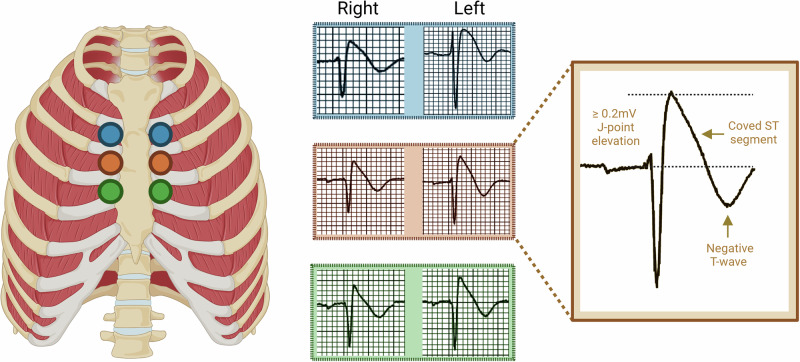
Demonstration of the recording of a high lead ECG with the precordial leads V1 and V2 placed in the second (blue), third (orange), and fourth (green) intercostal spaces. A type 1 Brugada ECG pattern is shown, which includes the hallmark findings of J-point and coved ST-elevation of ≥0.2 mV followed by a negative T-wave. Created in BioRender. Griffiths, R. (2025) https://BioRender.com/dv933lr.

The hallmark of BrS is the type 1 Brugada pattern which can be observed in the precordial leads (V1 and V2) of an ECG, placed either in the standard position (fourth intercostal space) or the ‘high lead’ positions (second or third intercostal spaces), as demonstrated in Fig. 1. This pattern consists of J-point and coved ST-elevation of ≥0.2 mV followed by a negative T-wave. The pattern may be identified spontaneously on a 10-second snapshot of a patient’s electrical activity (the ECG) or through a prolonged form of ECG recording made over 24 hours or more (Holter monitoring). A diagnosis of BrS is made in the presence of a spontaneous type 1 Brugada ECG pattern, once other diagnoses that mimic the pattern (known as phenocopies) have been excluded [1]. The type 1 Brugada pattern may also be unmasked through fever or on provocation challenge using a sodium channel blocker (e.g., ajmaline, flecainide, procainamide, pilsicainide). In such circumstances, a diagnosis of BrS should only be made if additional clinical criteria are met such as: a previous aborted cardiac arrest driven by pVT or VF; a family history of SADS in suspicious circumstances for BrS; a family history of BrS; or an episode of loss of consciousness that is felt to be suspicious for an underlying heart rhythm disturbance [1, 22]. Use of sodium channel blockers as a diagnostic test in otherwise healthy subjects without symptoms or family history can result in a false positive rate of 3% [23].

**Fig. 2:**
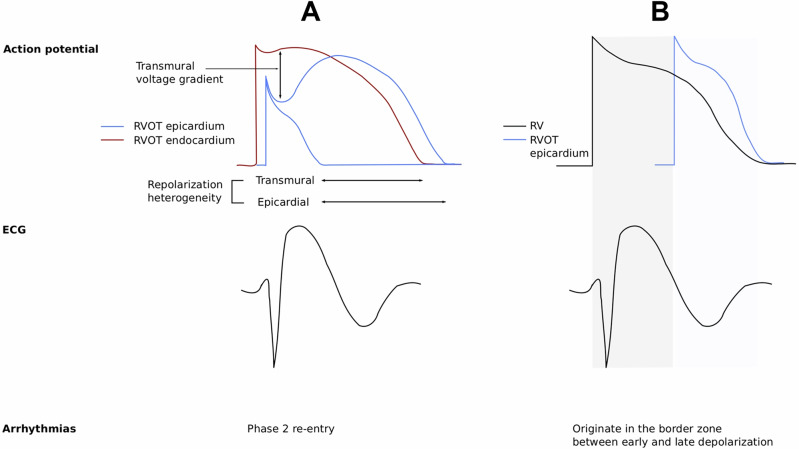
Electrophysiological theories underpinning the pathophysiology of Brugada Syndrome. The repolarisation theory (**A**) proposes that BrS results from a discrepancy of repolarisation across the right ventricular outflow tract wall, resulting from more unopposed transient outward potassium current via K_v_4.3. The alternative depolarisation theory (**B**) suggests that BrS results from delays in action potential depolarisation and structural changes in the right ventricular outflow tract. Please see the in-text description for more details. Figure reproduced, with permission, from Behr et al. [25] (license number: 6079250474677).

The pathophysiology of BrS is incompletely understood. Two theories have been proposed – the repolarisation and depolarisation hypotheses (Fig. 2) [24]. The former marks BrS as a disorder of repolarisation, where impairment of the inward current, including the sodium current (I_Na_), during the cardiac action potential causes more unopposed activity of K_v_4.3, the potassium channel responsible for the transient outward current (I_to_, Fig. 2A). This effect is more pronounced in the outer layer of the right ventricular outflow tract (RVOT) wall (the epicardium) than its inner layer (endocardium), causing an exaggerated and prolonged Phase 1 ‘dome’ of the action potential and thereby the characteristic type 1 Brugada pattern [24, 25]. In contrast, the depolarisation hypothesis originates from the observation that activation of the basal right ventricle (RV) and RVOT in patients with BrS occurs later than that without the condition (Fig. 2B) [26, 27]. The resultant voltage gradient set up between the RV body and the RVOT thereby causes the hallmark ECG appearance in the right precordial leads [24, 25]. Structural changes have also been observed in the hearts of patients with BrS, including fibrosis, inflammation, and diminished gap junction expression, which may contribute to the depolarisation disturbance [28–30]. It is possible that BrS results from a combination of these mechanisms leading to a cumulative impairment of ‘RVOT conduction reserve’ [25]. Underpinning these mechanisms is a patient’s genetic architecture.

## *SCN5A* – role in the development of the BrS phenotype

A genetic cause for BrS was first proposed in 1992, when two siblings with the classic type 1 Brugada ECG pattern both experienced aborted cardiac arrests at 2 years of age [19]. Chen and colleagues later proposed a link between BrS and *SCN5A*, the gene encoding the alpha subunit of the voltage-gated sodium channel in the heart, Na_V_1.5, important for the initial phase (Phase 0) of the cardiac action potential. This research group identified three rare variants (missense, frameshift, and splice-site) of *SCN5A* in six families and two patients with a sporadic form of BrS who displayed the classic type 1 Brugada ECG pattern [31]. The missense variant was shown to segregate in family members with VF and the type 1 Brugada pattern, which laid the groundwork for the hypothesis that BrS was an autosomal dominant disorder [31]. Functionally, the missense (p.T1620M) and frameshift (p.K1397Rfs*2) variants identified by Chen and colleagues were shown to confer a loss of function (LOF) effect on *SCN5A*, the former becoming increasingly apparent at higher temperature states [31, 32].

Rare coding variants in *SCN5A* are seen in approximately 20% of all BrS cases; however yield is much lower in those of Asian descent (up to 16% in Japanese populations, 8% in Taiwanese populations and 6% in Thai populations), despite an over-representation of BrS in these populations [11, 12, 33–37]. One explanation for the lower diagnostic yield is differences in genetic architecture. Walsh and colleagues recently demonstrated that a rare *SCN5A* intronic variant, located in the non-coding RE5 enhancer region, was enriched in Thai BrS probands [38]. Interestingly, this intronic variant accounted for a comparable proportion of BrS cases in the study population to disease-causing *SCN5A* coding variants in European populations, highlighting the differences in genetic architecture and propensity to BrS between ancestries [38].

Missense *SCN5A* variants account for the majority of genetically-explained BrS cases, causing disease through a variety of cellular mechanisms including altered gating and reduced expression of Na_V_1.5 or expression of defective Na_V_1.5 in the sarcolemma [12, 25, 39–44]. They are seen to cluster predominantly in pore-forming transmembrane regions of the gene [45]. Non-missense variants (i.e., LOF variants predicted to impair/eradicate the sodium current, including frameshift, in-frame deletion, copy number, splice site, and nonsense variants) account for approximately a third of BrS-causing *SCN5A* variants, leading to disease through the mechanism of nonsense-mediated mRNA decay and subsequent haploinsufficiency [46]. LOF *SCN5A* variants in BrS impair Phase 0 of the cardiac action potential and lead to slower conduction – this is more commonly seen in ultra-rare (gnomAD filtering allele frequency of less than 0.00001) than rare (gnomAD filtering allele frequency of between 0.00001 and 0.0001) variants [45]. Moreover, in a clinical study of patients with *SCN5A* LOF variants, those patients harbouring variants with the greatest functional effect (i.e., those resulting in aberrant protein truncation or a > 90% reduction in peak sodium current) had longer conduction intervals and were more likely to experience syncope [47]. The latter finding was the first to suggest a prognostic role of *SCN5A* variants in BrS.

## *SCN5A* – role in phenotypic severity in BrS

Patients with BrS harbouring a (likely) pathogenic variant in *SCN5A* (those who are ‘genotype-positive’) have been shown, across a range of ancestries, to have a more severe phenotype compared to those without (‘genotype-negative’).

Electrocardiographically, genotype-positive BrS patients are more likely to have conduction disturbances than those who are genotype-negative [48–50]. Moreover, genotype-positive BrS patients are more likely to present with a spontaneous type 1 Brugada pattern, the major electrocardiographic risk marker in BrS, and exhibit late potentials, a putative risk marker [49, 51]. Indeed, the likelihood of family members in *SCN5A* genotype-positive pedigrees exhibiting a type 1 Brugada pattern is also influenced by the biophysical severity of the variant. Subjects with truncating variants have a higher likelihood of showing the Brugada phenotype than those with missense variants. Patients with the most common pathogenic variant, *SCN5A-*E1784K, had the lowest risk of expressing disease [52].

Clinically, genotype-positive patients have been shown to have a poorer prognosis than those who are genotype-negative. Yamagata and colleagues studied 415 probands with BrS over a mean follow-up of 72 months across multiple sites in Japan [48]. 15% of the cohort had a disease-causing *SCN5A* variant; these probands experienced cardiac events (specifically, appropriate shock from an ICD and aborted or fatal cardiac arrests) more frequently and at an earlier age [48]. Similar observations have been found in Caucasian populations [49, 51], notionally recommending the presence/absence of a disease-causing *SCN5A* variant as a factor to be considered in BrS risk stratification.

The type and location of *SCN5A* variant may also play a role in risk. A recent study performed by Aizawa and colleagues on a small cohort of Japanese patients with BrS and functionally relevant *SCN5A* variants (defined as variants that result in less than 65% of the wild-type peak sodium current) found that non-missense variants in *SCN5A* led to a more severe phenotype (i.e., increased conduction delay and an increased risk of arrhythmic events) [46]. Moreover, Yamagata and colleagues observed that those with an *SCN5A* variant located in the pore region had worse outcomes than variants present elsewhere [48]. This evidence suggests that the type, location, and functional consequence of *SCN5A* variants may also need to be taken into account when considering risk in BrS.

## Non-*SCN5A* genetic influence

As LOF variants in *SCN5A* are only detected in around one-fifth of BrS patients, alternative candidate genes were explored to explain the missing heritability. These included LOF variants affecting the coding regions of sodium voltage-gated channel alpha subunit 10 (coded for by *SCN10A*), calcium channel subunits (*CACNA1C, CANB2*, *and CACNA2D1)*, sodium channel beta subunits (*SCN1B, SCN3B*), and proteins affecting expression and trafficking of sodium channels (*GPD1L*, *RANGRF, SLAMP*) [25, 53]. Gain of function variants in potassium channel genes have also been suggested, including those in the coding regions of voltage-gated potassium channel subunits responsible for the transient outward current (*KCNE3, KCND3, KCNE5*) and the ATP-sensitive potassium channel (*KCNJ8* and *ABCC9*) [25, 53]. The evidence in support of these alternative candidate genes initially led to their incorporation into clinical BrS genetic testing panels across the world [54].

Subsequent research however, cast doubt on whether they could be considered as causal variants. Pathogenic variants in these genes were noted to have a higher-than-expected prevalence in the general population for an autosomal dominant Mendelian disease [55]. Furthermore, rare coding variation in *SCN5A* was the only significant association with BrS cases compared to controls in an enrichment study of arrhythmia-susceptibility genes in those of European descent [56]. Such evidence led to a reappraisal of all genes previously implicated in BrS using the ClinGen gene-disease re-evaluation framework, by Hosseini and colleagues in 2018 [54]. This reappraisal consisted of three separate expert curation panels, which scored and combined available genetic and experimental (in vitro and in vivo) data, attributing a classification strength of definitive, strong, moderate, and limited for each gene to the development of BrS [54]. *SCN5A* was the only gene out of those assessed that was deemed to have ‘definitive’ evidence of causation of BrS, the remaining 20 genes were classified as showing ‘limited’ evidence of causation, thereafter downgraded to ‘disputed’ by an Expert panel [54]. The reappraisal concluded that clinical genetic testing in BrS should only include *SCN5A*, a recommendation that has been adopted into European Society of Cardiology clinical guidelines [1, 54].

## Challenges to the Mendelian paradigm

Since it was first suggested that BrS had a Mendelian (autosomal dominant) mode of inheritance, evidence challenging this paradigm has arisen. As previously stated, the yield of *SCN5A* variants in BrS is approximately 20% with alternative candidate genes proposed and later disputed following application of the rigorous ClinGen gene-disease re-evaluation framework [11, 12, 54]. However, the most compelling evidence to suggest a more complex genetic background in BrS is seen in genotype-positive families. Not only do variant carriers not always develop BrS (with incomplete penetrance estimates of around 30%), genotype-negative family members have been shown to develop BrS (a form of ‘genotype-phenotype mismatch’) [57, 58]. Taken together, these observations suggest a much more complex underlying genetic aetiology of BrS than was first believed.

## Contribution of common genetic variation

Common genetic variation may help to explain these observations. One example of this is the common single-nucleotide polymorphism (SNP) in *SCN5A*, H558R (c.1673 A > G), shown to have an allele frequency of 23% in those of European ancestry and less than 10% in those of Asian descent [59]. Since 2003, in vitro experiments have demonstrated that H558R can positively modulate *SCN5A* in the presence of LOF *SCN5A* variants, correcting the deleterious effect on the sodium current through restoration of normal channel gating kinetics or by rescuing trafficking defects [60–62]. This observed in vitro protective effect manifests electrocardiographically; BrS patients carrying both the H558R variant and a disease-causing *SCN5A* variant were shown to have more favourable ECG parameters than those without H558R [63]. Matsumura and colleagues subsequently compared BrS cases with and without the H558R genotype (heterozygous and homozygous) to controls [61]. Not only was the H558R variant observed more frequently in controls than in those with BrS, but those with BrS and the H558R variant had fewer episodes of VF over the follow-up period of 76 months [61]. This demonstrates how a common genetic variant can modulate phenotypic development and arrhythmic risk in BrS. These concepts have been explored on a larger scale by genome-wide association studies (GWAS).

## Genome Wide Association Studies (GWAS) – identifying the contribution of common genetic variation to the development of the BrS phenotype

Several GWAS have been performed to examine the contribution of common genetic variation to the development of BrS. Bezzina and colleagues performed the first of these in 2013, comparing 312 individuals of European descent with BrS to 1115 healthy controls [64]. This GWAS identified three BrS-associated ‘risk’ alleles, located at the *SCN5A* (rs11708996), *SCN10A* (rs10428132), and *HEY2/NCOA7* (rs9388451) loci. A cumulative risk score composed of the number of risk alleles at each of the three loci demonstrated that those carrying four or more were more than 20 times more likely to have BrS, than those carrying one or none [64].

The strongest association signal (rs10428132) was observed in the fourteenth intron of *SCN10A*, the gene coding for the alpha subunit of the sodium voltage-gated channel Na_V_1.8, located adjacent to *SCN5A* [64]. An abbreviated portion of *SCN10A*, composed of the final 7 exons of this gene (so-called *SCN10A-*short), is expressed in the heart and modulates Na_V_1.5 activity by increasing I_Na_ density, without affecting its gating properties [65]. It has been suggested that genetic variation within and surrounding *SCN10A* may affect the expression levels of *SCN10A-*short, thereby affecting I_Na_ through modulation of Na_V_1.5 [65] - lower expression of *SCN10*-short has been associated with a reduction in I_Na_ and higher expression associated with increased I_Na_ [65]. Indeed, *SCN10A*-short overexpression has recently been explored as a possible gene therapy for *SCN5A* haploinsufficiency and has been shown to reverse conduction slowing and prevent VT in in vitro and in vivo models [66].

An additional SNP identified in Bezzina and colleagues’ GWAS, rs9388451, was located close to two genes – *NCOA7*, a gene without previous links to cardiac function or disease, and *HEY2*, coding for a basic helix-loop-helix transcription factor which has previously been shown to be involved in cardiovascular system development [64, 67]. The candidate gene that was felt more likely to be responsible for the signal was *HEY2. HEY2* expression was shown to positively correlate with that of *KCNIP2*, a gene involved in the regulation of I_to_ and therefore important for maintenance of the normal electrophysiological gradient in the heart [68]. This offers an explanation for the increased risk of disease in carriers of the risk allele.

To expand on Bezzina and colleagues’ work, a larger genome-wide association meta-analysis in 2,820 unrelated individuals with BrS and 10,001 individuals without the condition of European ancestry was performed, identifying 21 independent SNPs across 12 loci that were associated with BrS, of which 10 loci were novel [69]. 8 independent SNPs were located at the *SCN5A/SCN10A* locus and 10 were observed in or nearby to genes coding for transcription factors involved in cardiac development, including *HEY2* [69]. An additional association signal was observed overlapping *MAPRE2* (rs476348), the gene coding for a member of the microtubule-associated protein RP/EB (MAPRE) family, which contributes to the organisation of microtubules [70]. rs476348 was associated with a reduction in expression of *MAPRE2* [69]. In subsequent functional modelling, LOF of *MAPRE2* resulted in dysfunction of microtubule structure and dynamics leading to altered trafficking of Na_V_1.5 to the cell membrane, as evidenced by a lower I_Na_ density, a reduction in velocity of the action potential upstroke in the ventricle, and a slower conduction velocity [69, 71]. These findings suggest another contributory mechanism to the development of BrS and showcase the use of GWAS in identifying novel genetic targets for functional exploration.

This GWAS, performed by Barc and colleagues, observed that a considerable proportion of BrS heritability was attributable to common genetic variation, with estimates varying from 0.17 to 0.34, depending on the method used [69]. Moreover, almost a quarter of this heritability was ascribable to the 12 loci in the study which had obtained genome-wide significance [69]. This finding highlights the considerable contribution of common genetic variation to the heritability of BrS, reinforcing its departure from the traditional Mendelian model of disease.

**Fig. 3:**
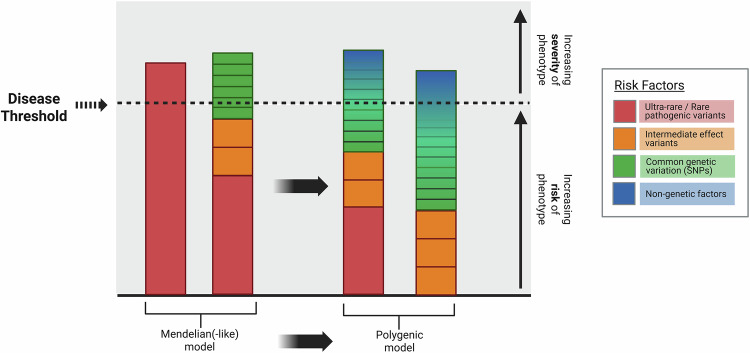
Genetic architecture of Brugada syndrome. Historically felt to be a monogenic disorder with an autosomal dominant (Mendelian) form of inheritance, BrS is now believed to result from accumulated genetic and environmental risk that surpasses a ‘disease threshold’ - the higher the accumulated risk, the more severe the clinical phenotype. Figure adapted from Walsh et al. (2020) [85] with permission (license number: 6079250358237) and created in BioRender (Griffiths, R. (2025) https://BioRender.com/gf3a16j).

A BrS polygenic risk score (BrS-PRS) was calculated, incorporating the 21 risk-conferring alleles and their corresponding effect sizes. Those with rare, disease-causing variants in *SCN5A* were shown to have a lower BrS-PRS than those without these variants (odds ratio of over 22 compared to 2.35 for four or more risk alleles), corroborating previous findings that, although the BrS-PRS increases the risk of developing the BrS phenotype in allcomers, its effect is greatest in those without an *SCN5A* rare variant [52, 69]. This observation may offer some explanation for the development of BrS in *SCN5A*-negative patients in genotype-positive families. It also lends credence to the idea of a ‘disease threshold’, whereby BrS develops as a result of accumulated genetic (and environmental) risk surpassing a ‘disease threshold’, with a higher accumulated risk resulting in a more severe clinical phenotype (Fig. 3).

**Fig. 4:**
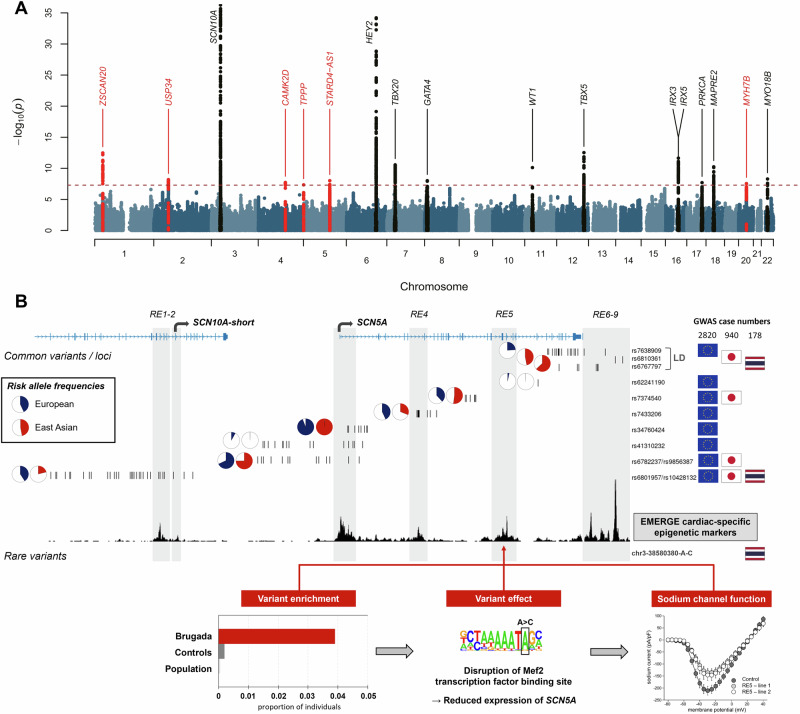
Brugada GWAS and non-coding variation at SCN5A/SCN10A locus. Panel (**A**) depicts the cross-ancestry Manhattan plot from Ishikawa and colleagues’ recent BrS GWAS with chromosomal position (1-22) depicted on the x-axis and the -log10 p values on the y axis [72]. Genome wide significance level is set at 5 × 10^−^^8^ and is represented by the horizontal dashed line. Loci are coloured red if they have not previously been identified in past GWAS and black if they are known. Reproduced with permission (license number: 6079250442604) [72]. **B** *Common variants/loci*: The location of eight conditionally independent loci at *SCN5A/SCN10A* from the European case-control GWAS (*n* = 2820 cases) are highlighted – lead SNPs plus other SNPs in strong linkage disequilibrium (LD, r^2^ > 0.5 using European samples). Replication of these loci in the Japanese (*n* = 940 cases) and Thai (*n* = 178 cases) GWAS is noted, with the risk allele frequencies in Non-Finnish European (blue) and East Asian (red) populations for the lead SNPs shown in the adjacent pie charts. The location of the SNPs is displayed with respect to the exons of *SCN5A* and *SCN10A*-short (blue), known promoter and enhancer regions (grey), and EMERGE cardiac-specific epigenetic markers (black). Of the eight loci, three were found at genome-wide significance in both European and Japanese studies (rs7374540, rs6782237/rs9856387, rs6801957/rs10428132). Two are not replicated in the Japanese dataset due to extremely low frequencies (MAF < 0.001) of the risk allele (rs62241190, rs41310232) while another is not replicated due to the high frequency (MAF > 0.99) of the risk allele (rs34760424), the latter highlighting a risk locus that is highly relevant for East Asians but not detectable with case-control studies in these populations. One locus (rs7433206) was not replicated (*p* = 0.37) in the Japanese study despite a high allele frequency (MAF = 0.31), which may indicate a false positive association or a different underlying causal locus. The associations for the final locus at the 3’ end of *SCN5A* and RE6-9 enhancer region are complex – genome-wide significant SNPs are detected in each GWAS, which are in low LD with each other (r^2^ = 0.05–0.15). Note, the limited number of associations/replication in the Thai GWAS likely reflects the low sample size and power (*n* = 178 cases). *Rare variants*: Data for a recently described non-coding rare variant in *SCN5A* is highlighted. This variant in the RE5 enhancer region is strongly enriched in Brugada cases from Thailand compared to population-matched controls and disrupts a conserved base in a Mef2 transcription factor binding site. CRISPR-Cas9 editing of the variant into hiPSC-derived cardiomyocytes demonstrated a reduction in *SCN5A* expression and sodium current density compared to isogenic controls.

A recent cross-ancestry GWAS performed in 2024 by Ishikawa and colleagues identified 17 loci to be associated with BrS, 6 of which were novel (Fig. 4) [72]. This research group also performed an initial GWAS in a smaller Japanese population. One novel locus shared by both analyses was *ZSCAN20* [72]. This gene is not expressed in the heart but in the testes [73]. The authors demonstrated that the risk allele (rs16835523) was associated with a higher expression of *AZIN2*, the product of which is involved in the generation of testosterone [73, 74]. The authors postulated that increased expression of *AZIN2*, driven by the risk allele, would lead to an increase in circulating testosterone, thereby increasing the risk of the BrS phenotype [72]. Of note, BrS sufferers of Asian descent are more frequently male than their European counterparts [35]; moreover, the *ZSCN20* novel locus had a higher odds ratio per risk allele in the Japanese population than in the population of European descent [35, 72]. This mechanism may help explain the male preponderance of BrS, particularly in those of Asian descent.

An additional SNP identified in Ishikawa and colleagues’ GWAS was rs6816233, a variant found in the eighth intron of the *CAMK2D* gene. This gene codes for the delta chain of Ca2 + /calmodulin-dependent protein kinase II (CAMKII). The rs6816233 SNP was shown to downregulate one form of CAMKII associated with pro-survival pathways and upregulate the alternatively spliced form, associated with pro-death and inflammatory pathways [72]. This pathway may help to explain the findings of fibrosis and inflammation in the hearts of BrS patients. This work again highlights the utility of GWAS not only in expanding our understanding of the genetic background of BrS and its impact on phenotypic development but also to provide targets for functional studies that help develop our understanding of the mechanisms underpinning BrS pathogenesis.

Common genetic variation has also been shown to play a role in the prediction of type 1 Brugada pattern development on provocation challenge using ajmaline [75]. Tadros and colleagues demonstrated that a BrS-PRS consisting of three SNPs modestly predicted the development of a type 1 Brugada pattern on provocation challenge, with predictive ability improving on incorporation of additional electrocardiographic and clinical details [75]. These findings suggest an emerging role for early assessment of common genetic variation, which may help to avoid unnecessary provocation challenges and their inherent limitations (such as side effects, adverse events, and limited availability) [75].

## Translatability of GWAS findings across ancestries

Ancestry differences in both BrS prevalence and diagnostic yield of *SCN5A* rare coding variants have previously been highlighted, with those of Asian descent being at a higher risk of developing BrS and less likely to have an *SCN5A* variant. GWAS in East Asian populations are therefore essential for the identification of ancestry-specific genes/loci and to ensure the translatability of PRS across ancestries [31, 70, 73].

Makarawate and colleagues sought to explore the contribution of common genetic variation to a Thai BrS population through an ancestry-specific GWAS [34]. Two haplotypes previously identified in the Bezzina and colleagues’ original GWAS were also identified in this study: rs10428132 (located within an intronic portion of *SCN10A*); and rs3734634 (located close to the *HEY2* locus and in close linkage disequilibrium with rs9388451) [34]. One association signal, rs6767797, at the *SCN5A/SCN10A* locus, was found to be novel [34]. These findings suggest at least some shared genetic risk between Thai and European ancestries, which was recapitulated in studies of Taiwanese and Japanese populations [64, 76].

Ishikawa and colleagues sought to explore shared genetic risk across ancestries by comparing the 17 lead SNPs identified in their GWAS across ancestry backgrounds (Fig. 4) [72]. 16 out of the 17 SNPs were demonstrated to have the same direction of effect across Japanese and European cohorts, with positive correlation evident in effect sizes and alternative allele frequencies [72]. Effect direction was also shared for 14 out of 17 SNPs in a Thai dataset, the comparisons limited due to sample size [72]. Building on this, Ishikawa and colleagues generated a modified BrS-PRS from Barc and colleagues’ 2022 GWAS, excluding 5 variants which were found to have a minor allele frequency of less than 0.01 (indicating they were not part of common variation in the Japanese populations) and applied this to their Japanese cohort [72]. The BrS-PRS was shown to associate with BrS in the Japanese populations, with an increasing score correlating with an increased risk of phenotypic development [72].

Overall, these studies provide some evidence that there are shared common genetic contributors to BrS across ancestries. However, differences in allele frequency, and effect size/direction may hamper the generalisability of a single BrS-PRS across ancestries.

## Genome Wide Association Studies – identifying the contribution of common genetic variation to the severity of the BrS phenotype

Barc and colleagues’ GWAS explored the relationship between their calculated BrS-PRS and the severity of BrS phenotype. Those with a spontaneous type 1 Brugada ECG pattern, a marker of higher arrhythmic risk in BrS, were shown to have a higher PRS-BrS than those with a type 1 Brugada pattern provoked by administration of a sodium channel blocker [69]. This finding has been confirmed across ancestries [73]. No relationship between the BrS-PRS and life-threatening arrhythmias was observed using the same BrS-PRS, the only genetic relationship was the presence of a rare *SCN5A* (likely) pathogenic variant [69, 73].

Subsequently, a study performed by Kukavica and colleagues found that a BrS-PRS composed of the 3 lead SNPs from the first BrS GWAS was independently associated with a higher risk of a life-threatening ventricular arrhythmia [77]. Indeed, event rates were found to be similar in those with the highest BrS-PRS and those with a rare (likely) pathogenic variant in *SCN5A*. These findings are in contrast to those observed by Barc and colleagues – this discrepancy may represent differences in phenotyping, outcomes, and follow-up in a single-centre study (as in Kukavica and colleagues’ work) compared to that performed in a larger, multi-centre study. Nonetheless, Kukavica and colleagues’ provides additional evidence to support the concept of accumulating genetic risk of unequal equity (i.e., one rare *SCN5A* variant compared to several common risk alleles) resulting in an equal severity of disease, as depicted in Fig. 3 [77].

## The role of low-frequency variation

Low-frequency *SCN5A* variants (defined as a minor allele frequency between 0.001 and 0.0001) may also contribute to the missing heritability in BrS [34]. Makarawate and colleagues demonstrated an over-representation of low-frequency *SCN5A* variants in Thai BrS cases compared to controls [34]. This observation was influenced by two main variants: R965C, observed in just under 5% of cases and 0.5% of controls; and A1428S [34]. Studies of R965C demonstrate that whilst it does not affect I_Na_ density, it can affect the sodium channel gating properties with a LOF effect [78]. These data suggest a modest functional effect that may perhaps be expected in view of the population frequency of this variant. Low-frequency variants have also been postulated to increase the susceptibility to a particular phenotype and play a role in disease modification [25, 34]. For instance, a compound *SCN5A* mutation found in a Chinese Han family consisting of R965C and R1309H, was found to have a much greater effect on sodium current than each variant individually [79, 80]. The recognition and identification of low-frequency variants is challenging clinically due to their relatively higher population frequencies, considered to be ‘too common’ to be labelled as (likely) pathogenic. Evidence does however suggest that they contribute to the genetic risk in BrS and may be particularly important in Asian populations where rare *SCN5A* variants are less commonly observed.

## Clinical Application of Genetic Testing

The paradigm of BrS as a monogenic disorder caused by a single mutation in *SCN5A* gene no longer holds true. Evidence suggests that its genetic architecture is much more complex, with phenotypic and severity development depending on the accumulation and interaction of both genetic and environmental factors (such as fever and sodium channel antagonists, see Fig. 3). A thorough appreciation of a patient’s genetic architecture is evidently important in the clinical management of such patients to assist with diagnosis, prognostication and protection of those at the highest risk. There are, therefore, several considerations one must make when applying these principles to clinical practice.

### *SCN5A* variant classification

*SCN5A* variant classification is based on American College of Medical Genetics and Genomics and the Association for Molecular Pathology (ACMG/AMP) criteria, published in 2015 [81]. These criteria combine weighted evidence from different fields of research to classify variants as pathogenic, likely pathogenic, likely benign, benign or as variants of uncertain significance (VUS). Missense variants, the predominant causative variant in BrS, present a challenge to this classification in view of the relatively high background rate of rare benign variants and difficulties in the prediction of their functional consequence [33]. Indeed, a reappraisal performed in 2019 of all the published *SCN5A* variants implicated in BrS found that only 17% of missense variants could be classified as (likely) pathogenic using the 2015 ACMG/AMP criteria [82]. This was due to the lack of family segregation evidence (as large affected pedigrees are rarely observed for BrS) and limited functional data (25%) – two of the critical evidence classes for the interpretation of missense variants.

Case-control analysis demonstrates that the majority of rare missense variants detected in BrS probands are likely to be causal, with ultra-rare variants in the transmembrane and pore regions of *SCN5A*/Nav1.5 particularly enriched in patients. A customised ACMG approach that incorporated quantified estimates of these hotspot enrichments led to reclassification of over 65% from variants of uncertain significance to (likely) pathogenic [33, 81]. While there may be reluctance to define variants as clinically actionable in the absence of specific evidence of pathogenicity, striking an effective balance between sensitivity and specificity in genetic testing remains a critical task.

High-throughput assays may help to circumvent the inherent challenges of missense variant interpretation in BrS. Functional data from an automated patch clamp assay, validated over two sites, was shown to accurately predict pathogenicity of *SCN5A* variants, classed as such by the 2015 ACMG criteria [83]. Building on this work, the assay was applied a BrS cohort – its functional outputs were able to reclassify almost 50% (*n* = 110 of 252) of VUS in the cohort [45]. Moreover, their study suggested that the greater the functional perturbation, the higher the penetrance of the variant [45]. Such assays provide robust functional data in a high-throughput manner, which can be applied directly into the interpretation of missense variants.

### *SCN5A* genetic analysis

Most clinical genetic test panels will only include coding regions of *SCN5A*, without full analysis of the non-coding regions of the gene (promoter, introns, enhancers etc.). This analysis would currently therefore miss rare non-coding variation in *SCN5A*, which may affect splicing or impact the regulation of the gene, which are emerging pathogenic variant classes for BrS (Fig. 4) [38].

### Polygenic risk score

As outlined, polygenic risk scores have utility in the prediction of both phenotypic development and severity in BrS. There are challenges to the incorporation of these scores into routine clinical practice:

- **Adjustment of genetic testing** – as previously mentioned, current clinical genetic testing for BrS consists of panel-based exon sequencing of *SCN5A* only. An effective BrS-PRS would require the use of an SNP genotyping array or whole genome sequencing – this comes at an additional cost. A cost-benefit analysis would need to be performed prior to incorporation into clinical practice.
- **Qualitative vs quantitative approach** – at present, the results of a clinical genetic test provide a binary outcome to explain the disease i.e., a disease-causing variant is either present or absent. A PRS would provide a more quantitative measure of risk – a very different approach. Additional training and expertise would be required for the interpretation of PRS results and their application to patients.
- **Development of the Polygenic Risk Score** – although there is some evidence from the aforementioned GWAS studies presented to suggest a cross-ancestry shared genetic risk of BrS, differences have been observed. This leads to difficulty in the generalisability of a single BrS-PRS to mixed populations and challenges in the practicalities of having separate BrS-PRS for each ancestry background.
- **Optimal timing of genetic analysis** – as discussed previously, PRS have shown utility for the prediction of response to ajmaline provocation challenge [75]. Classically, in clinical practice, genetic testing is performed following diagnosis of BrS and would not generally be performed prior to provocation challenge. The optimal timing of genetic analysis therefore requires careful consideration.
- **Discrepancies in event prediction** – as previously outlined, later GWAS studies incorporating more SNPs in their PRS were unsuccessful in their application of PRS in event prediction, but a study using three SNPs identified in the first GWAS was successful. Any clinically incorporated BrS-PRS would need to be accurate to avoid over- or under-implantation of ICDs, the current method of managing those patients at the highest level of risk [84].

Clearly, a greater understanding of BrS-PRS is required before they can be easily incorporated into general clinical practice.

## Conclusion

In conclusion, this review outlines the growing body of evidence for a polygenic aetiology for BrS. Current evidence supports the idea of a ‘disease threshold’ model, whereby BrS develops from accumulated genetic (and environmental) risk surpassing a ‘disease threshold’, with a higher accumulated risk resulting in a more severe phenotype. Ongoing large-scale whole genome sequencing studies, such as the 100,000 Genomes Project, that allow exploration of ultra-rare, rare, low-frequency, and common variation will help us to further our understanding of the genetic architecture of BrS. Whilst such studies are a promising and exciting area of future research, incorporation of such information into clinical practice presents inherent challenges that require careful consideration.

## Data Availability

Data sharing is not applicable to this article as no datasets were generated or analysed during the current study.
